# Assessment of water safety competencies: Benefits and caveats of testing in open water

**DOI:** 10.3389/fpsyg.2022.982480

**Published:** 2022-09-28

**Authors:** Tina van Duijn, Kane Cocker, Ludovic Seifert, Chris Button

**Affiliations:** ^1^School of Physical Education, Sport and Exercise Sciences, University of Otago, Dunedin, New Zealand; ^2^CETAPS EA3832, Faculty of Sport Sciences, University of Rouen Normandy, Mont-Saint-Aignan, France; ^3^Institut Universitaire de France (IUF), Paris, France

**Keywords:** aquatic skills, environment, ecological dynamics, cognitive psychology, validity, outdoor, skill learning

## Abstract

Drowning has been the cause of over 2.5 million preventable deaths in the past decade. Despite the fact that the majority of drownings occur in open water, assessment of water safety competency typically occurs in swimming pools. The assessment of water safety competency in open water environments brings with it a few difficulties, but also promises tremendous benefits. The aim of this position paper is to discuss the benefits and caveats of conducting assessments in open water environments as opposed to closed and controlled environments, and to provide recommendations for evidence-based practice. The first theoretical section discusses the effects of the environment and key variables (such as temperature and water movement) on various factors of assessment. These discussions are linked to the two perspectives of representative learning design (based on ecological dynamics) and information processing theory. The second section presents two pilot studies of relevance and provides practical implications for assessment of water safety competency. It seems that a combination of pool-based practice and open water education may be ideal in assessing aquatic skills competency. Assessment in open water presents clear benefits regarding validity, but often poses seemingly unsurmountable barriers, which providers may have reservations about in the absence of clear evidence. Hence this article provides a robust discussion about competency assessment and signals the practical importance of faithfully reproducing the environment in which skilled behavior is most relevant.

## Introduction

Drowning has been the cause of over 2.5 million preventable deaths in the past decade (UN General Assembly, 2021). It is responsible for more deaths than hepatitis or maternal mortality and close to that of malnutrition (World Health Organisation, 2022). The World Health Organisation (2021b) has made the provision of basic swimming and water safety skills a strategic priority for the next decade. In emergencies, such as drowning situations, skilled behavior can save lives, hence it is crucial that skills are assessed robustly (Chan et al., 2020). Skill assessment forms an integral part of any instructional program, so practitioners need scientific evidence base to inform assessment policy (Langendorfer and Bruya, 1995).

In general, the assessment of motor skill competency takes place in controlled and predictable environments where knowledge and skills are initially broken down and demonstrated in turn. For example, in a typical car driving test learners are first required to pass basic theory and visual function tests, then they are asked to perform some rudimentary driving maneuvers (e.g., emergency stop, reverse park, etc.), and finally they need to demonstrate they can drive safely in more realistic traffic conditions. Only once a rudimentary level of skilled behavior can be reliably demonstrated in controlled settings do practitioners go on to assess skill in more realistic (and challenging) natural environments. Relatedly, assessments of children’s fundamental movement skills (such as running, jumping, and throwing) are historically undertaken in the absence of play or game-related contexts (Ng and Button, 2018). For reliability and safety reasons one may appreciate why skill assessments are typically undertaken in such a way, but it is not well known whether such movement assessment batteries can discriminate amongst different levels of performance (Cools et al., 2009).

In this article, we focus attention on the assessment of water safety competency. There is clearly a need for evidence-based recommendations on instruction and assessment of these skills, and “[…] a more encompassing and dynamic view of water competence and drowning prevention education that addresses the dynamic and complex nature of drowning” (Stallman et al., 2017, p. 25). Consequently, we shall explain some important theoretical considerations from the motor learning perspective to help underpin the limited evidence currently available. Then, we go on to describe two case studies that illustrate some of the challenges of assessing skills in open water. The aim of this position paper is to discuss the benefits and caveats of conducting assessments in open water environments as opposed to closed and controlled environments (i.e., swimming pools or flumes).

## Background: Assessment of aquatic skills

**Figure 1:**
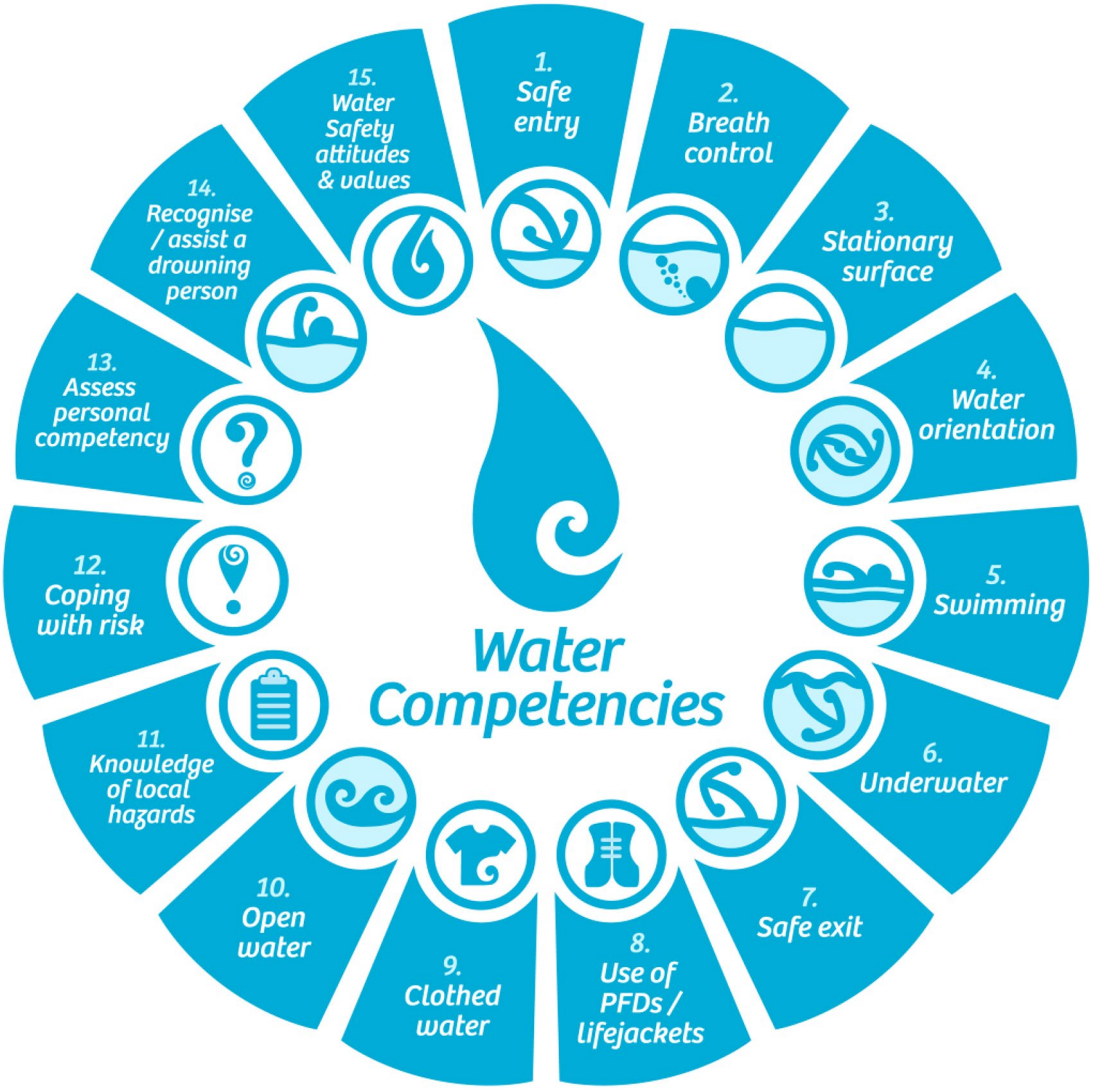
The 15 water competencies required to reduce the risk of drowning. Reproduced with permission of Drowning Prevention Auckland (https://www.dpanz.org.nz/courses/water-safety-for-children).

Aquatic skill competency is much more than being able to swim (Langendorfer and Bruya, 1995). Stallman et al. (2017) have proposed 15 different fundamental aquatic skills that form the basis of aquatic skill competency assessments, with swimming being only one of 15 competencies (see Figure 1). Robust assessments of water safety skills should include a range of competencies such as getting into and out of water safely, floating, breath control, underwater swimming, and recognizing hazards for oneself and others.

At least in developed nations, the assessment of swimming and water safety skills is typically undertaken in swimming pools, usually as part of education classes (Erbaugh, 1978; Moran et al., 2012; Stevens and NZCER, 2016; Di Paola, 2019; Chan et al., 2020). Swimming pools provide a seemingly “ideal” setting for competency assessments as the environmental conditions are relatively comfortable, stable, and reproducible (i.e., water temperature, currents, waves, depth, etc.). For example, Erbaugh (1978) showed that even when testing relatively unskilled individuals (i.e., 2–6 years old preschool children) it is possible to achieve high levels of inter-rater reliability when assessments are undertaken in a pool. However, introducing more variability in the water conditions of a swimming pool (such as waves) is likely to impact upon a learner’s aquatic abilities. Indeed Kjendlie et al. (2013), showed that when open water-like conditions (i.e., waves) are simulated in a pool, the levels of skill competency are markedly lower. In their study, 66 11-year-old children performed identical tests in two different environments: a calm swimming pool and a simulated wavy environment (30–40 cm amplitude). The tests consisted of a 200 m swimming time trial, a 3 min floating test, a diving entry to the pool, and a rolling entry. The tests performed in the waves clearly showed a performance decrement (between 9 and 14% longer time to complete the swimming test and 21, 16, and 24% lower scores for rolling entry, diving, and floating tests, respectively). Kjendlie et al. (2013), highlighted the fact that children “should not be expected to reproduce swimming skills they have performed in calm water with the same proficiency in unsteady conditions during an emergency” (p. 303). To our knowledge no studies have compared learning of adults’ performance of water safety-related competencies between different environments, and similarly with children the evidence-base is poor (Quan et al., 2015; van Duijn et al., 2021b).

Whilst swimming pools are the chosen location for swim lessons in most developed countries around the world, the majority of drownings happen outdoors (Quan et al., 2012). For example, out of 74 drownings in 2020 in New Zealand, the large majority (87%, *n* = 65) occurred in open water environments (Water Safety New Zealand, 2021). In the United States, open water drownings outnumber swimming pool drownings, because open water is the major drowning site for school age children, adolescents, and adults (Quan et al., 2008). In lower and middle-income countries, the main locations of drowning are ponds, lakes, rivers and ditches (World Health Organisation, 2014). As the range of external factors causing open water drowning (e.g., travel, work, flood-related disasters, etc.) is much wider than that of factors causing swimming pool drownings, the burden of open water drowning is much more difficult to quantify (World Health Organisation, 2022a).

To address the issue of assessment in naturalistic environments Hulteen et al. (2015), conducted a systematic review of field-based assessments for movement skill competency in lifelong physical activities. Only two published studies satisfied the inclusion criteria in relation to swimming or aquatic activities (Erbaugh, 1978; Zetou et al., 2014). In both studies, children under the age of 12 years were tested, and a swimming pool was used for the assessments. Whilst Erbaugh (1978) assessed several aquatic skills (water entry, front and back locomotion, breathing, kicking, underwater object retrieval), Zetou et al. (2014) only assessed backstroke. For both studies the inter-rater reliability of the assessed skills was high (Erbaugh, 1978: *r* = 0.89–0.99; Zetou et al., 2014: *r* = 0.79). Unfortunately, neither study considered the validity of the methods used nor whether the skills were as well produced in a different environment other than a swimming pool. To our knowledge this is the only published research investigating the effects of environment on the production of fundamental aquatic skills, and more evidence is urgently needed (Button, 2016). Furthermore, very little has been done to investigate the transfer of skills from controlled environments to real-life open water situations, which is arguably one of the biggest questions to be addressed in drowning prevention globally (Button and Croft, 2017; Guignard et al., 2020). Neither have the reliability and validity of aquatic skills assessment tools been tested with regards to predicting open water competence, risk of drowning or injury.

Summarizing thus far, water safety skills are typically assessed in swimming pools, yet they are arguably most required in open water (e.g., Lepore et al., 2015; Quan et al., 2015). Assessment of skills in pools may be misleading, as these skills are not assessed under the added pressure and mental stress that the natural environment can provide. Factors such as waves, currents, depth, visibility, temperature, submerged obstacles, surveillance, and many more set different environments apart. It would appear that water safety practitioners have generally neglected the critical issue of skill transfer to different aquatic environments. Parents and teachers may falsely assume that if their child can swim in a pool they are “drown-proof,” and likewise an increase in confidence by pool-trained swimmers may lead them to undertake more dangerous behaviors in the open water. On the contrary, according to the organization “Safe Kids Worldwide,” the assumption that *a child that is able to swim in a pool will be safe in open water* may be one factor contributing to global drowning statistics (Mackay et al., 2018). Di Paola (2019) comments that: “Many swimming and lifesaving programs, although well-structured on paper, lack valid and reliable skills assessment and verification, which in turn might lead to inadequate skills acquisition and development, to a false sense of safety and to over confidence in the water that, as we all know, can be extremely dangerous.” (p. 1).

## Theoretical considerations for the design of water competency assessment

Motor skill performance is influenced by the task and environmental context (Newell, 1985), which may be problematic in the realm of water safety skills assessment: a multitude of factors differ between closed, controlled environments, and open water. Given the multitude of influential constraints that shape motor behavior, it can be very difficult to maintain fidelity in assessment environments (see section 4: practical considerations). Depending on the basic theoretical standpoint from which one views motor performance, one might make different conclusions on how skill should be assessed in open water surroundings. The two main theoretical frameworks that are common in motor behavior research are the ecological dynamics framework and information processing theory. In this next section, we will map out the conclusions that can be drawn when viewing the issue from each theoretical standpoint.

### Information processing theories

Traditional information processing theories posit that programs stored within the central nervous system control muscle activation patterns for motor control. The organization of movement is seen as a top-down process driven by conscious processes and controlled in the cerebrum (Walsche, 1961). Skilled motor performance is viewed as an information processing activity guided by a general plan or program (Fitts, 1964). It may involve operations such as information translation, transmission, reduction, collation, and, most importantly, storage.

#### Information processing view on skill learning and assessment

The classical information processing model posits that information processing consists of three stages: stimulus identification, response selection, and response programming (Schmidt et al., 2018). During motor planning, a response (i.e., movement) and its control parameters (e.g., amount of force used) are selected and programmed, after which the motor command or program is executed *via* the motor cortex. During skill learning, relationships between control parameters such as speed and force and its outcomes, such as distance travelled, are learned (Schmidt, 1975, 2003; Sherwood and Lee, 2003). When we practice a motor skill that is relevant for water safety, we hope that this prepares us to cope with future situations – which are likely novel, unexpected and non-trained. Practice under more variable conditions is expected to accrue a more robust set of programs from which to inter-or extrapolate, and should therefore enhance learning (Boyce et al., 2006). Moreover, adaptation to novel situations (e.g., attempting to swim in waves) has also been shown to benefit from variable practice (Schmidt et al., 2018).

In variable environmental conditions, individuals have to identify and react to multiple, changing stimuli and select among a multitude of possible response options, while also adapting the control parameters of movement execution. Stimulus identification and response selection both become more complex. *Performing* in a more variable environment will therefore increase information processing demands and the chance of less-than-optimal response selection (Czyż, 2021), while *practicing* under such conditions may improve the future likelihood of succeeding in novel situations.

Based on recent efforts in computational and cognitive neuroscience (Clark, 2013; Koster-Hale and Saxe, 2013), a collection of models termed the *predictive processing framework* focus on the brain’s ability to predict the future (see Spratling, 2017 for an overview of theories within this framework): The framework posits that an internal model of the world, created on the basis of movements and past sensory experience, is used to predict future sensory input (Hawkins and Blakeslee, 2004; Körding and Wolpert, 2004; Friston, 2005; Spratling, 2010). Being able to predict the future is a considerable advantage to the human brain. Not only can we anticipate the sensory consequences of our own movements, but also the dynamics of objects and other agents in the world (Keller and Mrsic-Flogel, 2018). For instance, an ocean-experienced person may look at a photograph or video of an ocean wave and instantly predict what will happen next.

Internal models are learned – indeed, experience shapes the circuits required for generating predictions and computing prediction errors. Therefore, it is the interaction with the world that refines these connections to generate precise internal models. In the context of water competence, it is experience in different aquatic environments (be it ocean, beach or harbor), and interaction with natural elements such as rips, currents, rocks or murky water, that enable us to predict potential consequences, make safe decisions, and coordinate our movements appropriately. Sensory experience sculpts the connectivity between neurons in an activity-dependent manner, e.g., neurons that code similar responses become functionally linked into a network (Ko et al., 2011, 2013; Cossell et al., 2015; Keller and Mrsic-Flogel, 2018).

During learning of skills, errors invariantly lead to a deviation of the sensory input from the model-based prediction. These prediction-error signals are processed by the primary sensory areas of the cortex, updating the predictive model. Variability in the learning environment is therefore crucial to the development of a valid and broad predictive model – or in other words, errors may be necessary to learn the full range of what the consequences are possible in which environment: this speaks for a broad range of aquatic experience in a variety of environments. When assessing learning, only a situation with sensory consequences that provides the full complexity of a predictive model may accurately measure the adequateness or “fit” of the model and its level of advancement (Schmidt et al., 1987).

#### Working memory and decision-making

When learning motor skills, the learner tests hypotheses about how best to perform the skill (Magill, 1998). For this they use sensory feedback to assess the success of their actions (Bruner et al., 2017). This hypothesis-testing strategy generates a set of performance rules (declarative knowledge) that the learner may retrieve during practice and performance, until the movement has become automated and can be released from declarative control (Maxwell et al., 2003). Information that is manipulated during a motor task is held in working memory, a mental “workspace” with limited capacity (Baddeley and Hitch, 1974; Baddeley, 2012). Human working memory has a limited capacity: usually, a person may be able to store, process or manipulate 7 bits of information at the same time (Baddeley, 1994). This leads to interesting conflicts when a person has to complete several tasks, as is often the case in water safety emergencies. A drowning situation requires appropriate execution of movements (swimming, floating, treading water) alongside complex decision-making (e.g., swim to shore or save energy by assuming HELP (heat escape lessening posture)). When deciding on the correct behavior after an immersion, it is therefore important that cognitive capacities are available to evaluate the options and choose correctly. The more complex the environment and the more sources of information need to be considered for a decision, the higher the load on information processing resources (Logie, 2011). Making decisions in complex environments is therefore an overarching skill that changes in the face of a changing environment. Concurrent decision-making and cognitive secondary tasks have been shown to have a detrimental effect on motor performance (Poolton et al., 2006). Based on this viewpoint, we would therefore predict that performance of a motor task when assessed in open water would be lower compared to a pool environment.

During accidental immersion and other drowning incidents, panic and psychological stress are likely to occur. Similar to cold shock, panic leads to a cascade of physiological, cognitive, perceptual, emotional, and behavioral responses (Cameron et al., 1987; Murray, 2004), which may hamper motor performance and decision making in an emergency (Page et al., 2016). To address issues related to information processing overload, teaching methods that avoid high cognitive load have been suggested by proponents of information processing theory. For example, the use of implicit learning methods has been suggested in other fields (Masters, 1992; Hardy et al., 1996). Implicit motor learning refers to the acquisition of a skill in a non-verbal manner, with little conscious awareness of what is learned (Masters, 1992; Masters et al., 2004). This can be achieved by errorless learning, (i.e,, avoiding or minimizing errors during practice), or analogy learning, (i.e., an analogy is presented instead of declarative rules or instructions about the movement, e.g., “float like a starfish”). Studies using analogy in teaching swimming have shown that analogy instruction is effective for promoting efficient movement patterns (Komar et al., 2014, 2019). Focusing externally (on the effects of movements) may be a further strategy that benefits motor learning in a similar way (for a review, see Wulf and Prinz, 2001). Benefits of implicit and external focus-inducing instructions indicate that these may be powerful and cheap solutions to address the problem of information processing overload, however only two studies have used these approaches in water-related skills (Komar et al., 2014, 2019).

### Ecological dynamics theory

In contrast to information processing theories, the role of environment is central when one considers human behavior from an ecological dynamics theoretical perspective. How the environment is perceived in terms of opportunities to move, i.e., *affordances*, is a key idea from the ecological psychologist James Gibson. Gibson (1979) proposed that humans perceive objects, surfaces, or events by what they offer, invite, or demand in terms of action opportunities. Aquatic environment features such as waves and currents afford different actions for different people, due to, among other constraints, their distinct physical properties, such as their buoyancy (see Fajen et al., 2008; for key features of affordances discussed in the context of sport). According to Gibson (1979) perceiving the environment in terms of affordances renders dispensable those cognitive processes described above (Section 3.1) that transform action-independent perceptions into action-oriented perceptions. That is, in the process of direct perception, there is no integration and combination of cues involved.

Brunswik (1956) proposed the term *representative design* to advocate the study of psychological processes at the level of organism–environment relations. It means that perceptual variables should be sampled from the organism’s typical environment so as to be representative of the environmental stimuli from which they have been adapted, and to which behavior is intended to be generalized. The pedagogical principle of representative design ensures that the information-movement coupling of the structured practice environment is relevant and representative of the performance context (Pinder et al., 2011). What this means is that relevant information sources and affordances of the ‘to-be-learnt’ performance context should be present in a practice task (Button et al., 2020b). Ideally, such a practice task would not need to be a simulation of the real world, but rather a “sampling” of stimuli and affordances from the real context.

The importance of representative learning design may have particular significance when we consider the assessment of water safety skills. Guignard et al. (2020) suggest low skill transfer might be expected when people learn aquatic skills in a swimming pool versus in outdoor aquatic environments. To design representative performance (i.e., assessment) environments, practitioners should consider the following factors carefully. First, what are the interacting constraints on movement behaviors and how are they represented in the environment (i.e., action fidelity/realism). Second, it is crucial to adequately sample informational variables from the specific performance environments (i.e., relevant affordances) and thereby preserve the functional coupling between perception and action processes. Finally, practitioners should ensure that (i) the degree of success of a performer’s actions is controlled for, and compared between contexts (supporting transfer of skill and learning), and (ii) performers are able to achieve specific goals by basing actions (movement responses, decision making) on comparable information to that existing in the performance environment (Pinder et al., 2011).

In summary, both the information processing and ecological dynamics theories argue that practice in variable settings may lead to the development of more adaptable movement patterns that are better equipped to negotiate unpredictable demands (Reid et al., 2007). With regards to skills assessment, the ability to adapt one’s movement solutions to changing environmental demands is best assessed in situations that pose such demands.

## Practical considerations

In the following, we aim to provide some “food for thought” on the practical realization of water safety skills assessment. Although the focus is on assessment, most of these considerations may equally apply to the design of learning opportunities. The limited number of studies that have compared aquatic skills assessment between different environments forces us to rely on studies from different fields, theoretical considerations and preliminary data. In this light, we decided to include findings from two pilot studies, in the interest of driving future research efforts. Our priority was to explore potential difficulties associated with skills assessment in open water. Presumably, the paucity of research in this field may be related to worries about safety, limitations in available safety personnel and equipment, and access to safe outdoor environments. Case study 1 is a first attempt to determine whether water skill assessments in open water (1) are feasible and can be safely conducted, and (2) can lead to outcomes that are comparable to assessments in swimming pools. A second priority was to assess whether the use of an indoor flume (i.e., a pool through which a water current can be channeled at adjustable speed) could be an intermediate option for assessment of cardiorespiratory and physiological demands, as well as skill, during aquatic activities (specifically in this case, rescue). Since many physiological variables need to be assessed *via* advanced technological tools, it is – not least financially – near impossible to conduct such assessments in open water. If movement patterns (and, in future studies, physiological demands) could be replicated adequately in a controlled, indoor flume, this may open up avenues of skills assessment for the future (Pease, 1999). As such, case study 2 tells the story of a first attempt to simulate the full range of lifeguards’ movement patterns in a flume.

### Case study 1: Assessing children’s aquatic skills in open water and closed environments

#### Background

Button et al. (2020a) have recently explored different methods and environments to undertake a range of water safety assessments for young children. In this case study, we draw upon some of that data (which was collected in an indoor pool), and compare it with more recent data (collected in open water, van Duijn et al., 2021a). This will enable us to compare the outcomes of the same water safety skill assessment battery between the two environments.

#### Method

**Figure 2:**
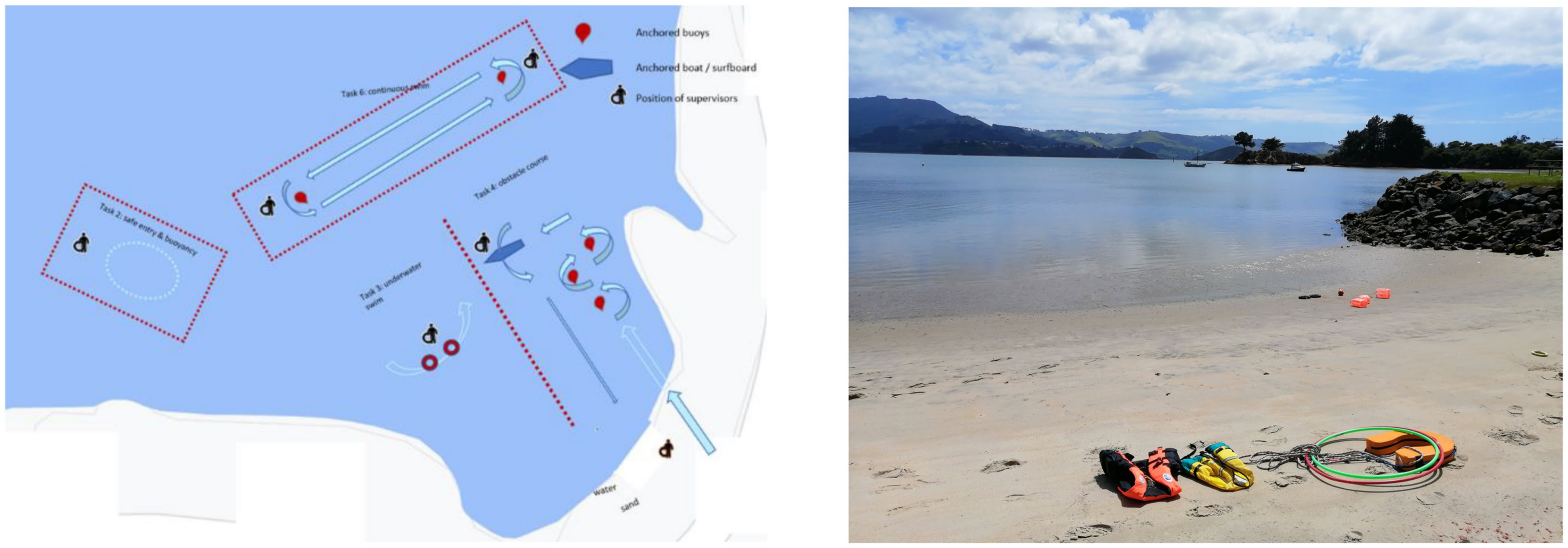
Experimental set-up for the outdoor testing sessions of the study. Left: Plan of the setup. Right: Overview of the beach testing location.

The indoor swimming pool that was used for assessments by Button et al. (2020a) was an 8 × 25 m rectangle, with a shallow (1.2 m) and deep end (2.5 m) joined by a continuous sloping floor. The pool had an access ladder in each corner and a support rail along each wall. For all testing sessions, the water temperature was set at 25°C. The open water environments were two similar beach reserves located in a harbor (see Figure 2). For the open water assessments, weather conditions were closely monitored and testing only proceeded in relatively settled conditions (i.e., ambient temperature + 13°C, wind <30 km/h). The swimming pool was booked for the purpose of testing and therefore not accessible by the general public but the open water environments were publicly accessible.

**Figure 3:**
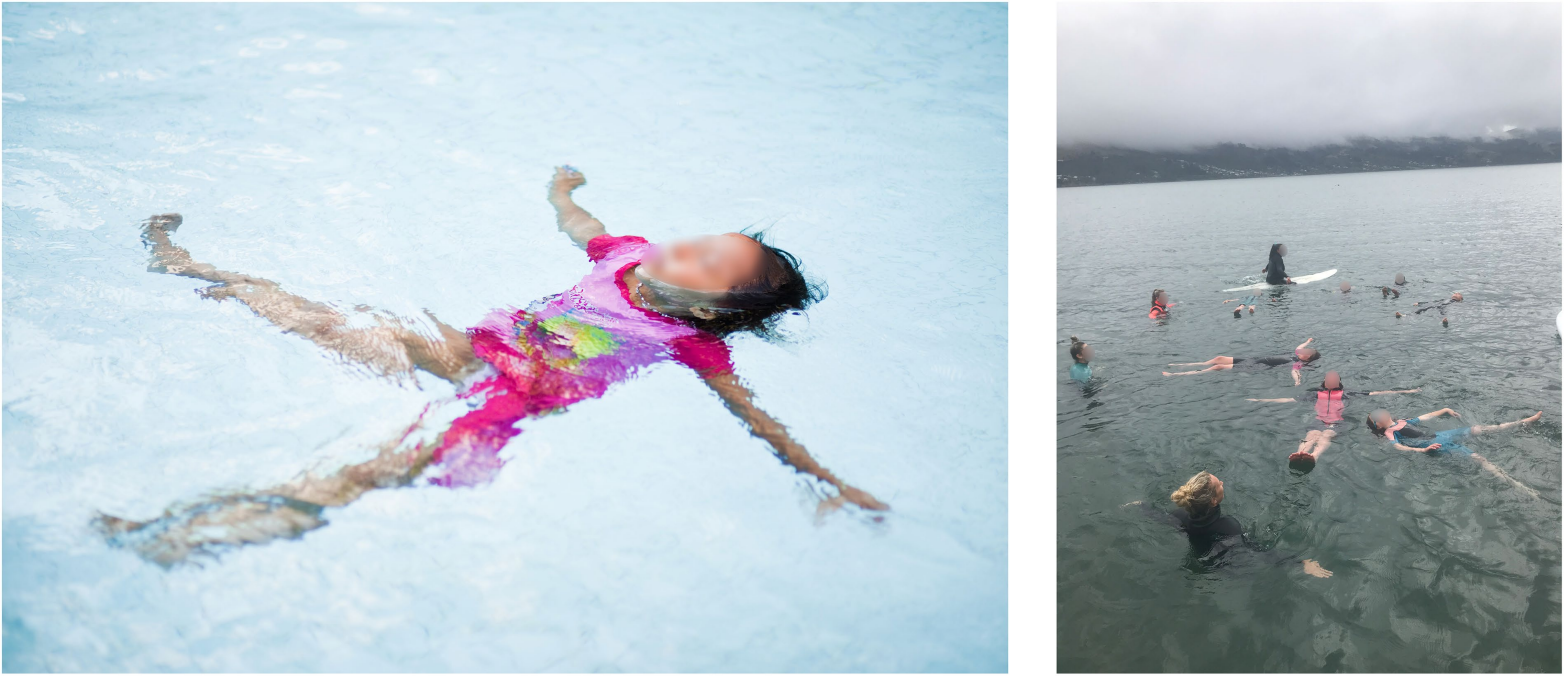
Impressions from the data collection: indoor testing in a pool (left) vs. outdoor testing in a harbor (right).

Two different groups of school aged children (5–13 years old) undertook four fundamental water safety skills assessments either in the swimming pool or harbor environments described above. There were 98 pool-tested children (44 female and 54 male) and 58 harbor-tested children (20 female, 38 male). The samples did not include complete novices (i.e., non-swimmers). The four tasks assessed were: a) floating and treading water (1 min of floating on the back as in Figure 3, followed by up to 4 min of treading water), b) completing an obstacle course, c) an underwater swim (surface dive and retrieve a submerged object 2–5 meters away) and, d) a continuous swim (swim using any stroke continuously for either up to 5 min or 100 m). All assessments (both pool and open water) were closely supervised by lifeguards and undertaken by experienced observers. Each task was visually assessed and graded on a 4-point scale.

#### Results

**Figure 4:**
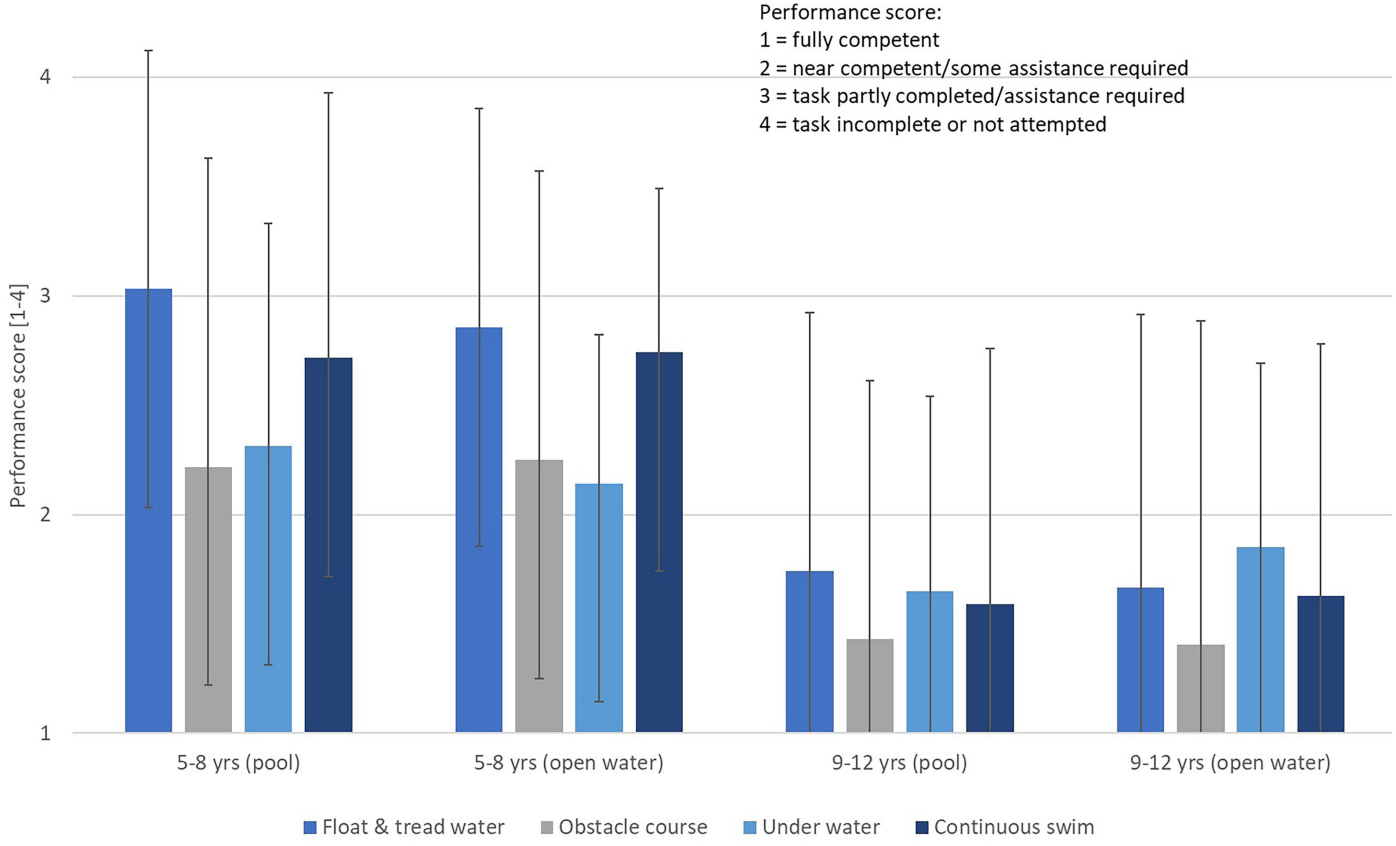
Comparison of water safety competency when children of different ages were assessed in a pool or in open water.

There were no notable or consistent differences in competency between pool and harbor environments. Independent sample Mann–Whitney U tests confirmed that there were no significant differences (*p* > 0.05) between competency scores in any of the four tasks. Regardless of the environment in which assessment occurred, older children (9–12 years) were more competent than younger children (5–8 years). One might have predicted the younger children (i.e., lower skilled) would be more nervous than the older children about being tested outdoors and hence their competency assessments would be reduced. However, the data did not support this prediction as younger children did not appear to perform worse in the open water tests than in the pool. The four skills seemed equally challenging for the children although the younger children showed a tendency toward lower scores in the floating/treading water and continuous swim tasks (Figure 4).

#### Discussion

An important component of water safety education is getting children to identify risks and how to manage them. This is very difficult to do in a swimming pool where the risks are different to outdoor aquatic environments. The fact that the open water assessments did not proffer different results from the study of Button et al. (2020a) imply that assessments of water competency may be successfully undertaken outside of a swimming pool. However, there were several challenges to overcome in terms of conducting skill assessments in a public harbor including ensuring adequate supervision, monitoring for boats and other water-users, and late cancelation of sessions due to poor weather. Assuming that such risks are managed appropriately it is possible to test water safety competencies in open water. Although younger children tended to perform less competently than older children (as would be expected), there was no strong evidence to suggest this difference is exacerbated when assessments take place in open water.

### Case study 2: Movement patterns used by surf life guards in open water and closed (laboratory simulation) rescues

#### Introduction

Typically, assessment of lifeguards’ swimming abilities has been conducted in closed, pool environments (Daniel and Klauck, 1992; Prieto Saborit et al., 2010; Salvador et al., 2014). However, reducing environmental complexity has been shown to alter the interaction between lifeguard and patient (or manikin, Avramidis et al., 2009), as well as gaze behavior and likely information processing (Seth and Edelman, 2004). Therefore, the external validity of pool-based lifeguard assessment may be questioned (Davids et al., 2006; Tipton et al., 2008; Holleman et al., 2020). As a compromise, flume environments allow to simulate some factors of an outdoor environment (e.g., current, waves, multitasking, longer distances without pause), while controlling the elements that would hamper valid and reliable data collection. Although flume testing has also been shown to alter swim technique when compared to indoor pools (Pease, 1999; Wilson et al., 2011; Espinosa et al., 2015; Guignard et al., 2017), a direct comparison with open water swimming has not yet been performed to our knowledge.

#### Method

**Figure 5:**
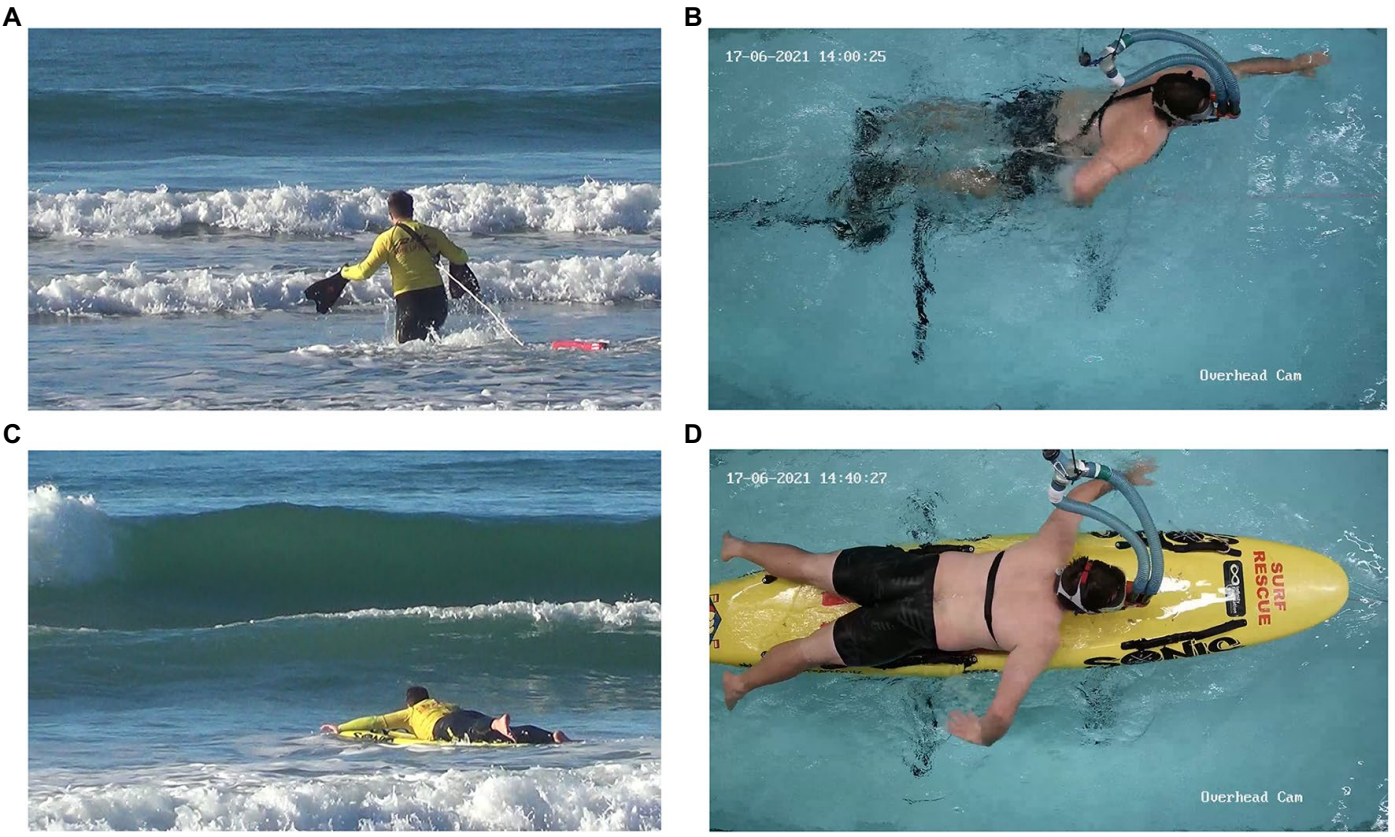
A lifeguard performing a tube rescue in open ocean **(A)**, a tube rescue in and controlled flume conditions **(B)**, a board rescue in open ocean **(C)**, and a board rescue in controlled flume conditions **(D)**.

This pilot study was conducted with five male experienced lifeguards (age: 16–51 years, experience 2–22 years). Two simulated rescues were performed by the lifeguards in a beach environment (open ocean, waves up to 3 ft) – one rescue by rescue board, and one by using a rescue tube and fins. Participants were asked to retrieve a manikin that was positioned in the water 100 m from shore as fast as possible. The type, order and duration of aquatic locomotion movements during each field test were subsequently described by an expert, and replicated step-by step in an indoor flume (see Figure 5 for a direct juxtaposition of the two environments): i.e., participants performed running, bounding, diving, swimming and paddling movements while their own video recording from their beach trial was played back to them. The speed of water flow in the flume was matched to the average speeds at which participants were moving during the beach trial. Based on expert categorizations, the type and duration of each movement pattern during each of the four tests was analyzed exploratively.

#### Results

**Figure 6:**
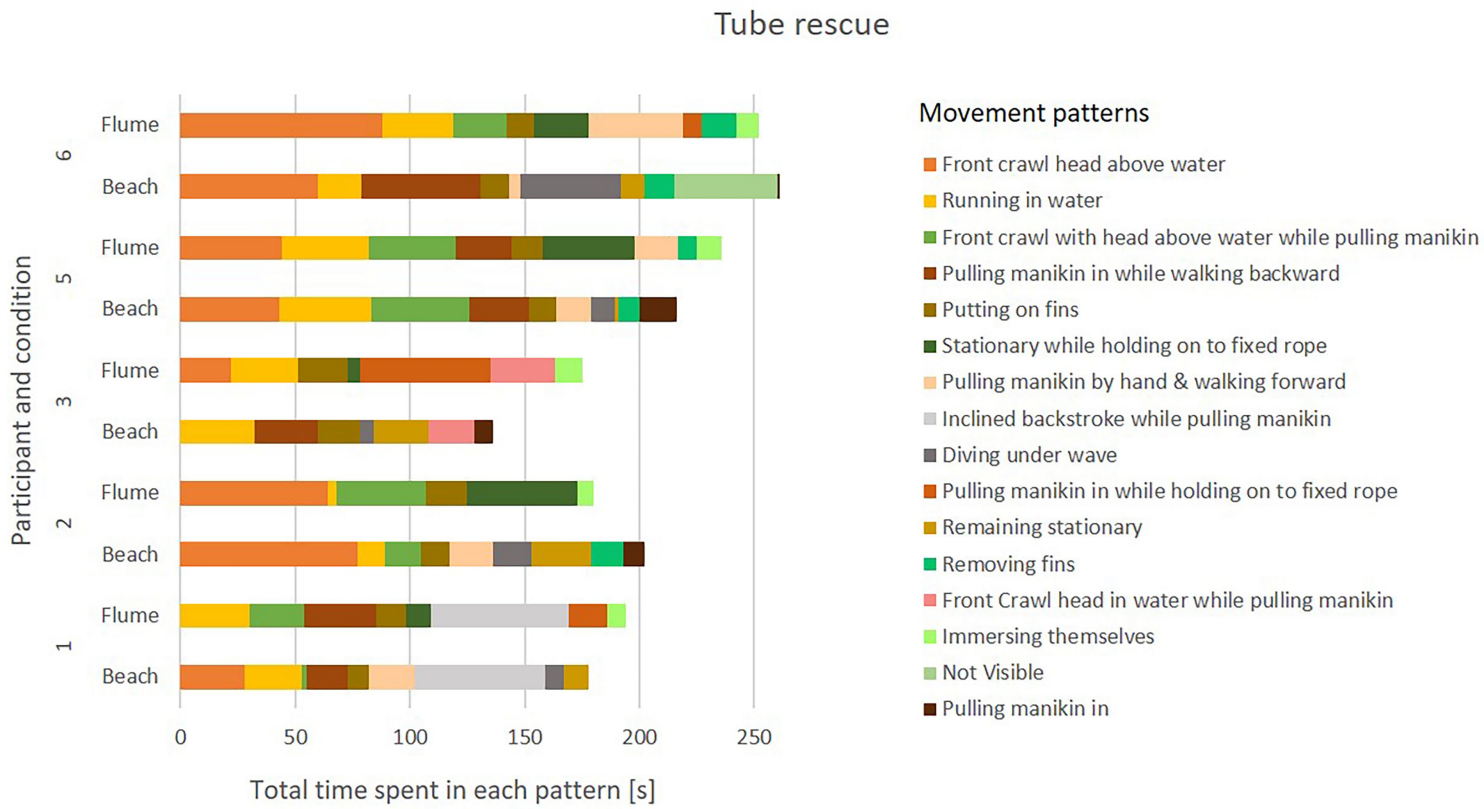
Movement patterns employed during tube rescue in ocean vs. flume conditions.

**Figure 7:**
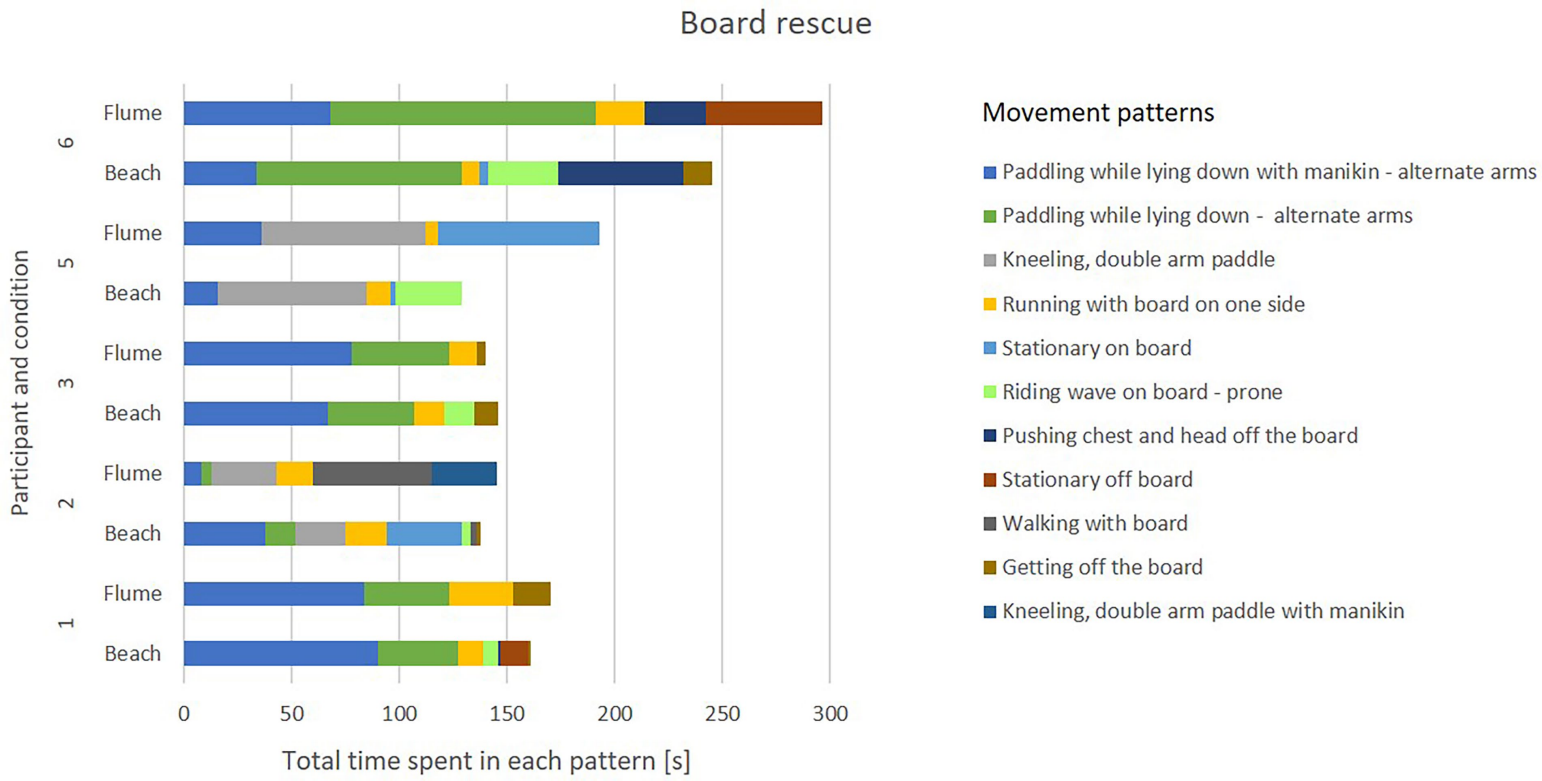
Movement patterns employed during board rescue in ocean vs. flume conditions. Note: The colors do not indicate the same patterns as in Figure 6.

During beach trials, participants employed a wide range of movement patterns (see Figures 6, 7). The relative time that participants spent in each pattern showed likewise large variations between participants. Within-participant comparisons of beach vs. flume movements suggest that the replication of movement patterns in the flume may have been overall successful, however, modifications due to the environment (water flow and pool depth) are clearly visible in the exhibited movement patterns. As an example, the pattern “diving under wave” was replaced by “immersing themselves,” a slightly adapted movement that mimics the former.

#### Discussion

Based on qualitative comparison of the movements, the replication of movement patterns in the flume seems feasible. However, it is likely that the flume replication may not have captured the intensity of the original movement, as participants reported finding it less taxing in the flume. Due to equipment limitations, it was not possible to fully replicate the “push back” resulting from waves, as well as some aspects of the original movements (e.g., walking in shallow water, overcoming breaking waves while fitting swim fins) and decision-making stimuli (e.g., timing, positioning, and patient status). Important aspects pertaining to the learner-environment interaction (perception-action coupling) may therefore have been missed in the simulation. If simulation fidelity is necessary for successful skill transfer to the real world, as suggested for example by Taber (2014), cognitive and psychological demands on the rescuer may also have to be simulated correctly.

#### Conclusion

This study was first attempt to replicate rescue movement patterns in a controlled flume setting. While flume-based simulations may allow correct replication of movement patterns, they are unlikely to represent the full range of demands (physical, cognitive, mechanical) presented when performing rescues in open water.

### Reflections on the type of competencies required to be safe in the water

As case study 2 shows, navigating open water environments may require perceptual and motor skills that are difficult to simulate in a controlled aquatic environment. It is clear that the characteristics of the environment dictate which skills are necessary to safely move around in it. When assessing skill, the relative importance that is attributed to different motor competencies should, in our view, depend on the environment in which the skill is predominantly applied. As an example, it is clear that immersion in cold water poses a direct threat to survival - either *via* the initial cold shock response involving gasping, hyperventilation, hypocapnia, tachycardia and hypertension, or subsequent hypothermia (Datta and Tipton, 2006). Immersion in cold water also affects people’s movements, rendering them stiff and inefficient. Thus, in a situation where a person falls into cold water, they need to have the knowledge that cold shock will cause them to gasp, have a high heart rate, and feel dizzy – but that it will subside after 2–3 min (Barwood et al., 2016). Such a situation would also require the person to be able to float first (i.e., for the time it takes for cold shock effects to dissipate) and then coordinate movement effectively to get out of the cold water quickly. In comparison, immersion in warm, but moving water might require a person to start swimming toward safety immediately, and would not require knowledge of cold shock. As a second example, many drownings or hospitalizations due to water-related injury have implicated clothing as an influential factor. Clothing likely impacts movement in the water *via* entrapment and by increasing the weight of the casualty (Keatinge, 1969), and thus also increases energetic, cardiorespiratory and cognitive demands on the swimmer (Choi et al., 2000; Stallman et al., 2011; Moran, 2014). Extending skills assessment to clothed swimming might be necessary to predict how well a person is able to cope in case of a realistic open water immersion incident. Whether a newly learned motor skill is transferred to performance in cold water and while wearing clothing has rarely been investigated (Schnitzler et al., 2017).

Another important component that defines the ability to move safely in aquatic environments is awareness of risks. Pitman et al. (2021) asked beachgoers to identify rip currents in photographs and *in situ* at a beach. They found that only 22% of respondents were able to identify the *in-situ* rip current, and of the respondents who correctly identified a rip current in photographs, 34% made correct *in situ* rip identifications. Furthermore, decision-making in aquatic environments heavily relies on accurate estimation of distance as well as one’s own motor and fitness and energetic capacities (Baird and Burkhart, 2000; Ducharme and Lounsbury, 2007). This highlights that perceptual performance may differ depending on the environment: photographs may not be the best means of teaching, nor of assessing the skill of identifying risk factors of the environment (e.g., Proffitt, 2006). The use of immersive and realistic simulations may be an avenue to explore in this respect (Baird and Burkhart, 2000; Ducharme and Lounsbury, 2007). Different culturally and socially developed habits, traditions and practices in relation to interaction with water are an important factor that needs to be pointed out here. For example, in bicultural New Zealand, a traditional Māori practice is food gathering in the ocean, through which a deep spiritual connection with nature is reached for, while recreational swimming in swimming pools is not very common. The colonial *Pakeha* population (European New Zealanders), by contrast, spend more time relaxing on the beach, surfing, or swimming for fitness in indoor pools (Phillips, 2020; Wheaton et al., 2020). With regards to assessment, this means that cultural factors should be considered when the ideal skillset for a person, in a given environment, is defined and assessed.

In summary, assessment of water competencies should differ depending on the physical and cultural environment under consideration, since the nature of the skills that are required to be safe depend on this environment. Due to the wide variety of open water locations, and thus of environmental constraints, assessment of water competencies needs to be situation-specific and tailored to the goal competencies that are deemed relevant.

### Reflections on optimal assessment tools in different environments

As we reflect on a wide range of complex issues, we must first acknowledge that perhaps the most important factor for assessing water safety competency that we have not discussed is the assessor themselves. They must be familiar with the appropriate behaviors to be demonstrated in each aquatic environment. For this reason, it is vital that experienced and trained practitioners undertake water safety assessments whatever the location. However, as we have explained at length in this article, the environment also dictates what is feasible and effective in regards to assessment.

In case study 1, the open water environments impacted how assessments were undertaken. It was discovered that using fixed buoys as reference points was problematic, as the changing tides led to ever-changing water depth. Fluctuating water conditions introduces various difficulties for testing such as increasing anxiety for those being assessed and potential exposing objects (e.g., rocks) that may have been covered in sand. As another example, while video recording may be applicable on a steep beach where it is easy to achieve an overview in a single frame, a river may not afford this way of assessing. Vice versa, monitoring testees on a boat may be more suitable in a river compared to a beach-break situation.

Measurement of water safety skills often involves assessing the maximal distance or time that a person can swim. Measurement tools to achieve this in open water could include a floating rope, buoys (useful in still water), a range finder (especially useful in waves, see case study 2) or a video recording with fixed points on land (e.g., in a river). The environment sometimes dictates specific clothing, which may in turn affect movement characteristics. A pertinent example is the wearing of wetsuits in cold water, which, although arguably necessary, changes the buoyancy of the learner, and their performance at certain tasks. Open water assessments are also impacted by waves and wind, which are hard to control and likely reduce the reliability of a testing scenario. Choosing a sheltered spot, though it reduces this risk, may alter a key constraint associated with the task, simplifying it and rendering it less externally valid. On the other hand, simulating elements of unpredictability that are common for open water contexts may be possible in pool settings: Perhaps using technologies such as wave machines, lazy rivers, and cold-water cannons in a pool may allow for a closer representation of open water features.

Assessment tools also need to include retention of skill over time and transfer of skills to more complex environments. One difficulty with delayed retention tests are changes in air and water temperatures with the changing seasons: a retention test at the end of summer will likely involve cold water (effectively rendering it a transfer test, see effects of cold water on motor skills described above). A transfer test is an ideal opportunity to test whether a child can cope with a novel scenario – ideally, it would assess whether the individual skills are being recalled, linked and executed in a realistic, outdoor setting. An example could be a simulated self-rescue which might involve surfacing, floating, treading water, navigating around objects and returning to shore. By assessing skill over a longer time scale, it would further be possible to confirm whether general transfer and learning transfer is improved (i.e., learn to learn, see Oppici and Panchuk, 2022). Additional to physical skills such as swimming, it may also be relevant to assess decision-making capability, which is even more tightly related to information that is available in the environment, and benefits from outdoor assessment. For example, a task “swim as far out as you can and come back” includes distance estimation, decision-making and estimation of maximal action capabilities. Such tasks can easily be adapted to open water, including waves and currents to enable assessment of the robustness of such skills to variable environments.

Clearly, assessment tools (i.e., skills and knowledge tests, or observational grading scales) also need to be tailored to the expected variability in skill level: a fine-grained scale that allows to discern small individual differences may be necessary in one situation, whereas in another the main goal might be to determine who can be unsupervised, and a rougher grading scale or single criterion may be used. This points toward the use of complex, multifactorial tests of movement and decision-making in a realistic environment to capture the person’s ability to cope in such situations. Transfer of each individual skill to a combined skill has rarely been tested in a water safety context (for an exception, see Button et al., 2022).

## Summary: Benefits and caveats of assessment in naturalistic environments

As we have previously highlighted, the assessment and learning of skills are closely intertwined. The variability and unpredictability of naturalistic environments would require self-organization by the learner. Additionally, this type of environment may favor perception-action coupling in a sense of a representative testing design, as learners will engage with the real context of performance. Naturalistic environments can help us educate to intention, i.e., to engage in task-goal oriented activity, involving searching strategy and decision-making especially in estimating risky places to swim vs. safe places to enter in the water and swim. For instance, being aware of low/high tide times would change the intention of learners, as a beach could be safe because sandy and flat at low tide, whereas this same beach could be dangerous because rocky with steep slopes at high tide. Thus, naturalistic environments would require that a learner explores their various possibilities of action (and consequently make decisions), such as where to enter and exit; in comparison to a swimming pool where safe access is more obvious (i.e., ladder vs. edge of the pool). Furthermore, naturalistic environments help to educate to attention, i.e., to attune to relevant information for action. For example, instead of learning what a risky situation is from photographs and simulation in a swimming pool (which arguably represent the structural properties of an environment), learners are invited to perceive functional properties of the environment. This process supports the perception of affordances (opportunities for action) relative to the learner’s own action capabilities.

More broadly, the naturalistic environment requires the learners to select, within a rich landscape of affordances, the action that best fits their action capabilities and intention. For instance, entering clear water (i.e., ground is visible) does not afford the same actions as does blurry water, because potential seaweed and rocks may change what types of locomotion are best suited. When the ground is visible, a learner might jump into the water whereas when it is not, they might use water shoes or walk smoothly into the water. Hence, whether the aim for the practitioner is learning or assessment of water safety skills, there is potentially much to be gained from utilizing representative environments.

As summarized in Table 1, the numerous benefits of assessing in naturalistic environment are accompanied by caveats. For example, difficulties arise in maintaining high reliability, as practitioners must control the variability and unpredictability of naturalistic environments. Replicability of a testing scenario is also important to allow comparisons between different kinds of naturalistic environment. A sharper focus on skill transfer (rather than skill reproduction) also seems necessary which may require assessment in multiple open water environments. We recommend that practitioners should carefully weigh up such benefits and caveats in designing water safety assessments in naturalistic environments.

**Table 1:** Benefits and caveats of skills assessment in open water.

Aspect	Benefits of assessing in open water	Caveats of assessing in open water
Quality of assessment	High external validity	Need careful designing to enable high construct validity – often, skills are not clearly separable Replicability requires precise definition of all parameters
Assessment of competencies beyond motor skills	Accurate simulation of information processing load Adaptability of the skill can be assessed accurately Perception-action coupling is similar to what is required in open water Accurate representation of relevant information for decision-making assessment	Difficulty to separate motor skills from other aspects
Individual differences	Individual differences in open water skill may be adequately shown	High variability makes consistent testing difficult. Need for a fine-grained assessment scale and for splitting skills into sub-components
Skill transfer	Best way to assess transfer of skill into relevant environment	Difficult to separate skill learning from transfer
Cultural relevance	Possibility to include wide range of practices and forms of interaction with the environment	
Safety	Possible to conduct safely in appropriate environment	Requirements at schools often extreme: need for high ratio of supervisors: learners Requirement to choose predictable environment reduces value of open water testing
Time efficiency	–	Requires more time to plan and run

## Conclusion and future research

In this article we have argued that water safety skill assessments must be carefully designed to reflect the range of competencies that may be required in naturalistic environments. Aquatic competency involves much more than just swimming. Assessment batteries that separate specific testable skills seem necessary to reflect the range of behaviors that may be required to remain safe in and around water. Contemporary motor learning theories support the inclusion of task and environmental variability to show how robust the performer is to variations that are common in natural aquatic environments. However, future research is needed to:

- Determine the effects of environment on the production of fundamental aquatic skills.
- To investigate the transfer of skills from the controlled environments to real-life open water situations.
- To assess the reliability and validity of aquatic skills assessment tools with regards to predicting open water competence, risk of drowning or injury.
- To find out whether, or to what extent, simulation of psychological and physiological demands is feasible in controlled lab-environments.

We have provided initial evidence that assessment in open water is possible, especially if the outdoor environment can be managed appropriately. There are important caveats that practitioners must carefully weigh up when designing assessment activities however, in our opinion the many benefits to be gained from testing in naturalistic environments outweigh such concerns.

## Author contributions

TD contributed to study concept and design (case study 1 and case study 2), acquisition of subjects (case study 1), data collection (case study 1 and case study 2), and preparation of the manuscript. CB contributed to study concept and design (case study 1 and case study 2), acquisition of subjects (case study 1), data collection (case study 1 and case study 2), analysis and interpretation of data (case study 1), and preparation of the manuscript. KC contributed to study concept and design (case study 2), acquisition of subjects (case study 2), data collection (case study 1 and case study 2), analysis and interpretation of data (case study 2). LS contributed to interpretation of data and preparation of the manuscript. All authors significantly contributed to the research process. All authors contributed to the article and approved the submitted version.

## Funding

This study was funded by the Swiss National Science Foundation (P2SKP1_187632). Water Safety New Zealand provided financial support for the two studies that are discussed in case study 1 (16/033 and 20/094).

## Conflict of interest

The authors declare that the research was conducted in the absence of any commercial or financial relationships that could be construed as a potential conflict of interest.

## Publisher’s note

All claims expressed in this article are solely those of the authors and do not necessarily represent those of their affiliated organizations, or those of the publisher, the editors and the reviewers. Any product that may be evaluated in this article, or claim that may be made by its manufacturer, is not guaranteed or endorsed by the publisher.
